# Comment on Cancer Metabolism and on the Role of the Endocrine Pancreas

**DOI:** 10.14740/jocmr1912w

**Published:** 2014-09-09

**Authors:** Maurice Israel

**Affiliations:** 2 Av. Aristide Briand 91440, Bures sur Yvette, and CNRS Gif sur Yvette 91190, France. Email: mauisrael@wanadoo.fr

## To the Editor

In a previous work published 4 years ago, an arrest of tumor cell proliferation was obtained with dichloroacetate, an inhibitor of pyruvate dehydrogenase kinase, aiming to dephosphorylate and activate pyruvate dehydrogenase (PDH) [1]. This observation indicates that it may be useful to act as well, on other tumor cell enzymes that change in parallel to PDH, since this leads to a “rewiring of metabolic pathways” that is specific for tumor cells. Altered signaling controls that inhibit, or activate, enzymes in tumor cells were recently reviewed [2, 3]. Schematically, in tumor cells, enzymes such as PDH and pyruvate kinase (PK) are phosphorylated and inhibited as observed for catabolism and gluconeogenesis, while other enzymes (for example, glycogen synthase) are dephosphorylated, in their anabolic configuration, associated to glycolysis. This hybrid situation rewires metabolic pathways in tumor cells and gives them a metabolic advantage, enabling them to plunder tissue reserves mobilized by catabolic hormones [4, 5]. Normally, pancreatic beta cells secrete in parallel to insulin, the transmitter GABA, which switches off neighboring alpha and delta cells releasing respectively glucagon and somatostatin. In this way, when anabolic insulin is released, catabolic glucagon is switched off by GABA [2]. A failure of this GABA mediated mechanism probably explains the hybrid catabolic/anabolic response of tumor cell; however, we also have to consider that differentiated cells in cancer, respond preferentially to catabolic hormones; this is discussed in this letter.

### Hormonal control of catabolism or anabolism

The mobilization of tissue reserves in starvation is a known physiological situation; catabolic hormones, glucagon and epinephrine, act on Gs-coupled receptors activating protein kinases (PKA/Src) that elicit the phosphorylation of downstream enzymes supporting the production of glucose by glycogenolysis and gluconeogenesis, while other activated enzymes, mobilize fatty acids forming ketone bodies. A typical feature of catabolism is a phosphorylation of PK and PDH that inhibits these enzymes, closing the entry of the Krebs cycle, which spares oxaloacetate (OAA) at the start of the glucose synthesis pathway.

In contrast, an increased glycemia elicits the release of insulin and other anabolic hormones. In anabolism, glycolytic metabolism takes over, oriented by the increase of its fructose 2-6 bis phosphate activator. New molecular building blocks are synthetized; cells can divide. The action of insulin and other anabolic hormones is mediated by tyrosine kinase receptors, controlling glycolysis, and the activation of MAP kinase and PI3 kinase signals for mitosis and cell survival. The selection switch between anabolism and catabolism is tightly controlled in the endocrine pancreas. When beta cells release anabolic insulin in response to glycemia, they switch off with a parallel release of GABA, alpha cells releasing catabolic glucagon, and delta cells also release somatostatin. GABA switches off alpha and delta cells via GABA A ionotropic receptors; the influx of Cl^-^ hyperpolarizes these cells, blocks the Ca^2+^ influx and the release of glucagon and somatostatin.

### A pancreatic GABA failure explains cancer metabolism

What would be the consequences of a failure of this GABA-dependent regulation of the endocrine pancreas? It may occur if glutamate decarboxylase (GAD), the GABA synthetizing enzyme is inhibited by pesticides (glyphosate), for example. The consummation of betel nuts (the second worldwide addiction after tobacco) is known to inhibit GABA-ergic processes, via nipecotic acid and favors cancer. We also suspect that vitamin B_6_ deficiencies affect GABA synthesis, since B_6_ is the cofactor of GAD. Such deficiencies might be provoked by compounds forming adducts with vitamin B_6_ aldehyde (hydrazines, amines, pyrroles), for example, isoniazide or gyromitrine from gyromitra mushrooms, in several cases there was a carcinogenic effect. Finally, autoimmune diseases affecting GAD have been described; it is the case for a diabetes type 1, Stiff-Person syndrome, or Paraneoplastic cerebellar degeneration. In this type 1 diabetes, beta cells are usually lysed, but weaker antibody might simply decrease GABA release. In the two other diseases mentioned the heavy neurological symptoms are frequently associated to tumors. All these possible causes that decrease the effect of GABA or its release from beta cells would alter the switch-off mechanism for glucagon that will then be released, together with insulin. A hybrid catabolic/anabolic message would be sent to cells; and indeed, tumor cells display a dual hybrid response. This is shown by the blockade of PK and PDH as for catabolism, paradoxically associated to a very active glycolysis and citrate condensation, feeding synthetic processes as for anabolism. To overcome the PK and PDH blockade, tumor cells rewire the system, they use for their citrate condensation, acetyl CoA coming from ketone bodies provided by tissue reserves, while OAA is formed via phosphoenol pyruvate carboxy kinase or malate dehydrogenase. Below the citrate condensation, a new stop in the Krebs cycle favors the citrate efflux from mitochondria, which starts via ATP citrate lyase and acetyl CoA carboxylase the synthesis of fatty acids and lipid membranes for mitotic cells; malonyl CoA closes the entry of fatty acids in mitochondria, rendering tumor cell dependent of ketone bodies and lysolipids sources [3]. Why then enzymes of differentiated cells, hepatocytes or adipocytes, respond preferentially to catabolic hormones; as if, in cancer, differentiated cells, were relatively insensitive to insulin signals. In a way, this resembles to a situation found in diabetes type 2, in which there is a desensitization of insulin receptors, interiorized by endocytosis before proteolysis. Insulin resistance is often associated to chronic inflammation with elevated cachexin; in diabetes type 2, there is indeed an elevated cancer risk [6]. For avoiding the insulin desensitization process, beta cells turn off insulin release, terminating its action. In this case, the released GABA acts on metabotropic GABA B receptors of beta cells that are coupled to Gi proteins. This closes after several steps the insulin release mechanism [7, 8]. Hence, GABA release from beta cells functions as an autocrine inhibitor, turning off insulin release. If GABA release gets deficient there will then be a persistent release of insulin, which desensitizes insulin receptors on differentiated cells that will respond preferentially to catabolic hormones. In mitotic cells, the situation is very different, since they respond to both insulin and glucagon, adopting a hybrid anabolic/catabolic rewiring. The simplest explanation is that in contrast to differentiated cells, submitted to insulin receptor desensitization, new stem cells express new insulin receptors that have not been desensitized. And since these cells have also Gs-coupled receptors, they will display the dual anabolic/catabolic response and start the rewiring process. Moreover, in adrenal medulla, a deficient GABA release stimulates epinephrine release, which inhibits somatostatin. Glucagon also stimulates cortisol release.

### Conclusion

The three catabolic hormones mobilize reserves from differentiated cells with desensitized insulin receptors. This is beneficial to new mitotic stem cells, which respond to both insulin and catabolic hormones, and rewire their metabolic pathways in a “cancer mode”.

Stem cell mitosis is triggered for compensating the loss of differentiated cells, why anabolic hormones and insulin are released? The repair process follows the disruption of differentiated cells, releasing their contents (sugars, amino acids) in the blood; these are sensed as “food intake” by beta cells that secrete insulin, mitotic signals are sent to stem cells. If there is a pancreatic GABA deficiency, a hybrid anabolic/catabolic message rewires metabolic pathway in stem cells, while differentiated cells resistant to insulin respond to catabolic hormones. Epigenetic changes stabilize the metabolic situation, and then mutations, presumably on an upstream protein kinase, are sufficient to stabilize the phosphorylation status of downstream enzymes involved in the rewiring process. Now, mutated cells escape from the systemic hormonal mechanism that started the process. Whether or not genetic mutations stabilize these metabolic pathways that are typical of tumor cell, there are several weak points, rendering tumor cells specifically dependent for given substrates [3, 5]. Indeed, reactivating PK, PDH, and inhibiting other up-regulated enzymes (ATP citrate lyase), may reset the system, reversing a hybrid situation favorable to stem cells and then to tumor cells. Finally, cancer prevention might require a surveillance of the endocrine pancreas.
